# A Novel Prognostic Factor TIPE2 in Bladder Cancer

**DOI:** 10.3389/pore.2022.1610282

**Published:** 2022-03-21

**Authors:** Yu Jiang, Zhiqiang Zhang, Xian Wang, Zhenzhong Feng, Bo Hong, Dexin Yu, Yi Wang

**Affiliations:** ^1^ Department of Urology, Second Affiliated Hospital of Anhui Medical University, Hefei, China; ^2^ Department of Pathology, Second Affiliated Hospital of Anhui Medical University, Hefei, China; ^3^ Anhui Province Key Laboratory of Medical Physics and Technology, Institute of Health and Medical Technology, Hefei Institutes of Physical Science, Chinese Academy of Sciences, Hefei, China; ^4^ Anhui Provincial Institute of Translational Medicine, Hefei, China

**Keywords:** epithelial-mesenchymal transition, bladder cancer, TIPE2, cystectomy, urothelial carcinoma

## Abstract

**Objective:** We sought to identify tumor necrosis factor (TNF)-alpha-induced protein 8-like 2 (TIPE2/TNFAIP8L2) expression in bladder cancer and its relationship to clinicopathological findings and prognosis.

**Methods:** Immunohistochemical (IHC) staining for TIPE2 was performed on 110 archived radical cystectomy specimens. Ten high-power fields were randomly selected from each specimen to observe and record the percentage of immunoreactive cells of TIPE2 in tumor cells (grade 0–4) and the corresponding immunostaining intensity (grade 0–3). The expression score of TIPE2 was obtained by multiplying the results of the above two scores, which ranged from 0 to 12 points. The cut-off point of the sum of the scores were defined as follows: 0–3 scores were defined as negative expression (-); >3 scores were classified as positive expression, < 7, low expression, ≥7, high expression.

**Results:** In 110 cases, TIPE2 was stained in various degrees in bladder cancer tissues, and expressed in both nucleus and cytoplasm. 4.5% (5/110) showed negative expression, 40.9% (45/110) showed low expression, and 54.5% (60/110) showed high expression. TIPE2 expression was negatively correlated with lymph node metastasis (*p* = 0.004) and disease progression (*p* = 0.021). Survival curves were plotted to show that patients with high TIPE2 expression had a progression-free survival curve above those with negative/low TIPE2 expression (*p* = 0.027). In multivariate Cox proportional hazard regression analysis, TIPE2 was a protective factor for progression-free survival in bladder urothelial carcinoma (*p* = 0.031), pT stage (*p* = 0.016) was a risk factor for progression-free survival, and age was a risk factor for overall survival (*p* = 0.020).

**Conclusion:** TIPE2 may be a new biomarker to predict the disease progression and prognosis of patients with urothelial carcinoma of the bladder.

## Introduction

Bladder cancer is the 10th most common malignancy worldwide and one of the most common malignancies of the urinary system [1-2]. According to data from the American Cancer Society’s statistical Annual Report 2020, bladder cancer ranked 4th in estimated new cases and 8th in estimated deaths in men [1]. Statistics from China Tumor Registration Center in 2015 showed that the incidence of bladder cancer was 8.05/100,000 and the mortality was 3.29/100,000, with an increasing trend year by year [3]. Bladder cancer has become one of the major threats to people’s health. Bladder urothelial carcinoma is the most common histological subtype of bladder cancer. In recent years, despite rapid progress in the diagnosis and treatment of bladder cancer, the mortality rate remains high [4]. Patients are prone to recurrence or metastasis after surgery, so finding an effective biomarker for diagnosis and treatment of bladder cancer is one of the urgent problems to be solved in clinical practice.

TIPE2 (TNFAIP8L2) belongs to the tumor necrosis factor (TNF)-alpha-induced protein 8 (TNFAIP8/TIPE) family, which is composed of 184 amino acids with an N-terminus death effector domain (DED) [5]. TIPE2 was initially found to be a negative regulator of innate and adaptive immunity through Toll-like receptor (TLR) and T cell receptor (TCR) signaling pathways. Subsequent studies have found that TIPE2 is also involved in multiple signaling pathways of tumorigenesis and development, and plays a key role in different processes of cancer cell survival, proliferation, migration and invasion [6, 7]. Among different cancer types, TIPE2 expression level was decreased in liver cancer [8], small cell lung cancer [9], stomach cancer [10], esophageal cancer [11] and prostate cancer [12], and increased in kidney cancer [13], colon cancer [14] and thyroid papillary carcinoma [15]. TIPE2 has shown its potential as a novel tumor therapeutic target and biomarker in various cancers. However, the expression condition and role of TIPE2 in the development and progression of bladder urothelial carcinoma are largely unknown.

In the present study, we demonstrated for the first time that TIPE2 was expressed in various degrees in bladder urothelial carcinoma tissues by IHC staining method. We also found that TIPE2 expression was negatively correlated to lymph node metastasis and disease progression of bladder cancer patients.

## Materials and Methods

The study was conducted in accordance with the Helsinki Declaration (revised in 2013). This study was approved by the ethics committee of the second affiliated hospital of Anhui Medical University (No. YX2021-109) and the application for exemption of informed consent for this retrospective analysis was approved.

### Clinical Data

From July 2018 to October 2020, a total of 142 patients underwent radical cystectomy combined with pelvic lymph node dissection for bladder cancer in the Urology Department of the Second Affiliated Hospital of Anhui Medical University. We excluded 9 patients with non-urothelial carcinoma, 7 patients with other tumors such as prostate cancer, 4 patients who had received neoadjuvant chemotherapy before surgery, and 12 patients who lost follow-up data. The remaining 110 patients had urothelial carcinoma and did not receive preoperative systemic chemotherapy or radiotherapy. Formalin-fixed paraffin-embedded tissue blocks (FFPE) of surgical specimens of bladder cancer from 110 enrolled patients were collected from the Pathology Department of the Second Affiliated Hospital of Anhui Medical University. All included patients had been pathologically confirmed and clinicopathological information was basically complete. There were 93 males and 17 females, with an average age of (67.0 ± 11.2) years. Among the 110 patients, 78 cases were newly diagnosed with bladder cancer and 32 cases were recurrent bladder cancer. There were 58 smokers and 52 non-smokers. The maximum diameter of the tumor was 0.8∼5.2 cm with an average of 3.3 cm. Pathological grading was performed according to WHO (1973) bladder cancer grading system [16], and TNM staging criteria of UICC(2017) bladder cancer [17] was used for bladder cancer staging. There were 26 cases of low grade urothelial carcinoma and 84 cases of high grade urothelial carcinoma. TNM staging: Ta 11 cases, T1 30 cases, T2 36 cases, T3 22 cases, T4 11 cases; N0 78 cases, N1 7 cases, N2 16 cases; M0, 109 cases, M1, 1 case. Overall survival time (OS) refers to the time from the end of surgery to death. Progression-free survival (PFS) was defined as the time from treatment initiation to disease progression, relapse, or death of any cause, whichever occurred first. The follow-up ended in November 2021, with a median follow-up of 22.5 months (range, 1.0∼40.0 months). Outpatient or telephone follow-up was used. Clinical data and follow-up data of all enrolled patients were complete.

### Experimental Methods

#### Preparation of Formalin-Fixed Paraffin-Embedded Tissue Block Sections

Eligible paraffin blocks from enrolled patients were selected and sequentially sectioned with 4 μm thickness. After section preparation, one was used for immunohistochemical staining, the other two were used for hematoxylin-eosin staining (HE) and blank control, respectively.

#### Main Reagents

TIPE2 Polyclonal Antibody was purchased from Thermo Fisher Scientific (Catalog # PA5-38711, RRID AB_2555305, 1:100 dilution). Immunohistochemistry universal detection kit (mouse/rabbit polymer detection system PV 6000) and DAB Chromogenic kit (ZLI-9019) were purchased from Beijing Zhongshan Jinqiao Company, China.

#### Immunohistochemistry and Score

The prepared paraffin sections were deparaffined, hydrated and repaired with sodium citrate buffer under high pressure. Hydrogen peroxide was dropped onto the slides and placed at room temperature for 10 min to remove endogenous peroxidase, then washed with PBS solution. One slide of each group was added with appropriate working concentration of TIPE2, and the other slide was added with PBS solution instead of primary antibody as blank control, and incubated at 37°C for 60 min. The primary antibody was rinsed with PBS solution, and the secondary antibody was dropped onto the slide. After incubation at room temperature for 20 min, the secondary antibody was rinsed with PBS solution. DAB chromogenic solution was added for staining, and the staining degree was observed. After reaching an appropriate depth, the staining was immediately washed with PBS solution, and the staining was finished. Finally, the slides were re-stained with hematoxylin and sealed. The staining results of slides were interpreted by two senior pathologists respectively. When the interpretation results were inconsistent, a third pathologist was asked to interpret and make the final judgment. All slides were semi-quantitatively scored by the double-blind method. Ten high-power fields were randomly selected from each specimen to observe and record the percentage of positively stained cells of TIPE2 in tumor cells (0: 0%; 1: 1∼25%; 2: 26∼50%; 3: 51∼75%; 4: 76∼100%) and the corresponding staining intensity (0: no staining; 1: light yellow; 2. Medium tan; 3: dark tan) [15]. The expression score of TIPE2 was obtained by multiplying the results of the above two scores, which ranged from 0 to 12 points. The cut-off point of the sum of the scores were defined as follows: 0–3 scores were defined as negative expression (-); >3 scores were classified as positive expression, < 7, low expression, ≥7, high expression.

#### Statistical Analysis

Data were analyzed using SPSS 24.0 (IBM, Armonk, NY, United States). The quantitative data of normal distribution was presented in the form of “mean ± standard deviation”. For the non-normal distribution and the quantitative data with no exact value or unclear distribution at both ends, the median (range, minimum ∼ maximum) was presented. According to the expression of TIPE2, age, gender, tumor TNM stage, pathological grade and recurrence of bladder tumor were divided into groups as dichotomous variables. Chi-square test or corrected Chi-square test was used for comparison between groups. The Kaplan-Meier curve was used to estimate the association between TIPE2 expression and survival (including PFS and OS), and the log-rank test was performed. Cox proportional risk regression was used to analyze the risk factors affecting the prognosis of patients. *p* < 0.05 was considered to be statistically significant.

## Results

### Relationship Between TIPE2 Expression and Clinicopathological Features in Bladder Cancer Patients

**FIGURE 1:**
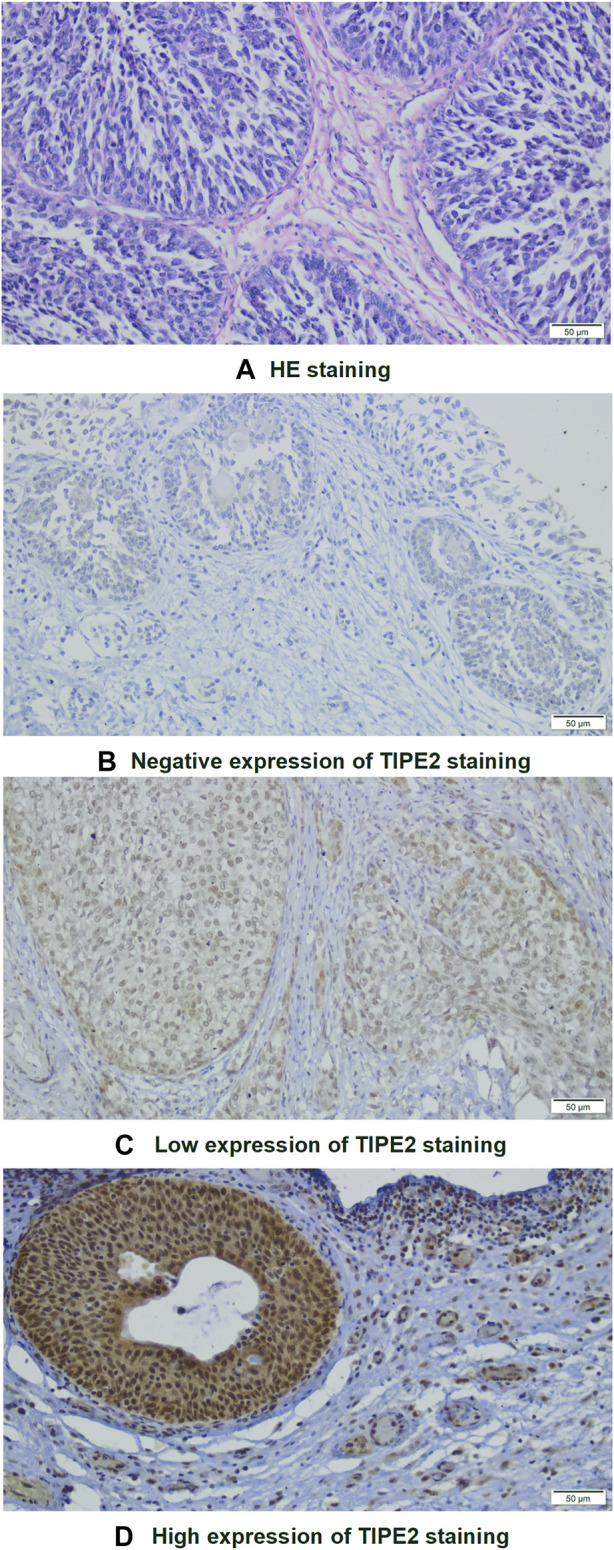
Images of HE and immunohistochemical staining of bladder tumor cells (×200): **(A)** HE staining. **(B–D)** Immunohistochemical staining of TIPE2: **(B)** Negative; **(C)** Low expression; **(D)**: High expression.

Firstly, the relationship between clinical characteristics and TIPE2 expression was analyzed. Hematoxylin-eosin (HE) staining was performed for identifying tumor areas (Figure 1A). Immunohistochemical (IHC) staining of TIPE2 in 110 cases of bladder cancer after postoperative pathological paraffinized tissue sections showed that TIPE2 was stained in different degrees in bladder cancer tissues and expressed in both the nucleus and the cytoplasm. After scoring, 4.5% showed negative expression (5/110, Figure 1B), 40.9% showed low expression (45/110, Figure 1C) and 54.5% showed high expression (60/110, Figure 1D).

The clinicopathological features of 110 patients and their relationship with TIPE2 expression were shown in Table 1. The expression of TIPE2 was negatively associated with lymph node metastasis (*p* = 0.004) and disease progression (*p* = 0.021), while age, gender, recurrence status, smoking condition, tumor T stage, vascular invasion, nerve invasion and pathological grade did not differ significantly between patients with negative/low and high TIPE2 expression.

**TABLE 1:** The relationship between TIPE2 expression and clinicopathological features.

	No. of patient (%)	TIPE2 expression		P^a^
		Negative/Low	High	
Total (%)	110	50 (45.5)	60 (54.5)	
Age				
≤65	46 (41.8)	21 (45.7)	25 (54.3)	
>65	64 (58.2)	29 (45.3)	35 (54.7)	0.972
Gender				
Male	93 (84.5)	43 (46.2)	50 (53.8)	
Female	17 (15.5)	7 (41.2)	10 (58.8)	0.700
Recurrent bladder cancer				
Yes	32 (29.1)	14 (43.8)	18 (56.3)	
No	78 (70.9)	36 (46.2)	42 (53.8)	0.818
Smoking				
Yes	58 (52.7)	22 (37.9)	36 (62.1)	
No	52 (47.3)	28 (53.8)	24 (46.2)	0.094
pT stage				
≤pT1	41 (37.3)	18 (43.9)	23 (56.1)	
≥pT2	69 (62.7)	32 (46.4)	37 (53.6)	0.801
Ta	11 (10.0)	4 (36.4)	7 (63.6)	
T1	30 (27.3)	14 (46.6)	16 (53.3)	
T2	36 (32.7)	16 (44.4)	20 (55.6)	
T3	22 (20.0)	10 (45.5)	12 (54.5)	
T4	11 (10.0)	6 (54.5)	5 (45.5)	0.943
Pathologic grade				
Grade 1 and 2	26 (23.6)	9 (34.6)	17 (65.4)	
Grade 3	84 (76.4)	41 (48.8)	43 (51.2)	0.204
Lymph node status^b^				
N0	78 (77.2)	31 (39.7)	47 (60.3)	
N1 or N2	23 (22.8)	17 (73.9)	6 (26.1)	0.004
Vascular invasion^c^				
Yes	52 (54.7)	25 (48.1)	27 (51.9)	
No	43 (45.3)	23 (53.5)	20 (46.5)	0.600
Nerve invasion^d^				
Yes	38 (40.9)	16 (42.1)	22 (57.9)	
No	55 (59.1)	29 (52.7)	26 (47.3)	0.314
Disease progression				
Yes	32 (29.1)	20 (62.5)	12 (37.5)	
No	78 (70.9)	30 (38.5)	48 (61.5)	0.021

a χ2 test or χ2 test of continuity correction.

b Nine patients had an unknown pathological status of the lymph nodes.

c Fifteen patients had an unknown pathological status of vascular invasion.

d Seventeen patients had an unknown pathological status of nerve invasion.

### Relationship Between TIPE2 Expression and Prognosis

**FIGURE 2:**
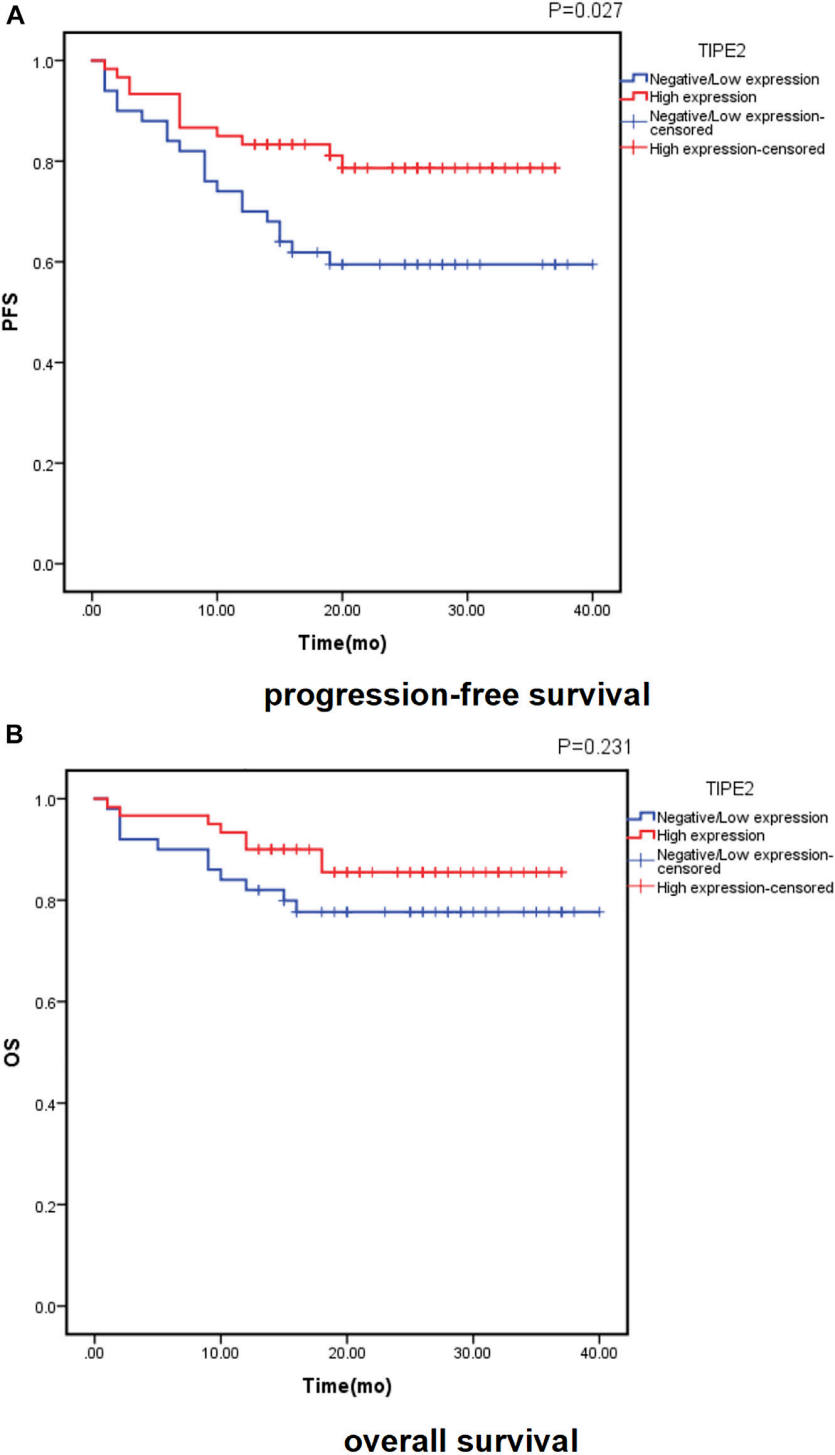
Probability of survival in patients with urothelial carcinoma of the bladder according to TIPE2 expression estimated using the Kaplan-Meier method: **(A)**: progression-free survival; **(B)**: overall survival.

Of the 110 patients, 32 patients (29.1%) had disease progression, with a median time of 7 months (range, 1∼20 months). At the time of analysis, 19 patients (17.3%) died, and the time of death was (8.7 ± 5.8) months. Follow-up data of 110 cases was recorded and analyzed for Kaplan–Meier survival curves (Figure 2). Both the PFS and OS survival curve showed that the survival rate of patients with high TIPE2 expression was longer than that of patients with negative/low TIPE2 expression. The difference in PFS survival curve was statistically significant (*p* = 0.027), although the difference in OS survival curve was not statistically significant (*p* = 0.231) between patients with negative/low and high TIPE2 expression.

Cox proportional hazard regression analysis was performed on the factors that might affect the progression-free survival and overall survival of bladder cancer. The results of univariate Cox regression analysis were shown in Supplementary Table S1: TIPE2 expression (*p* = 0.033) was a protective factor for progression-free survival of bladder urothelial carcinoma, while pT stage (*p* = 0.004), age (*p* = 0.023) and lymph node status (*p* = 0.033) were risk factors for progression-free survival of bladder urothelial carcinoma. Age (*p* = 0.006) and pT stage (*p* = 0.025) were the risk factors for bladder cancer postoperative overall survival. TIPE2 expression, pT stage and age were first included in the multivariate Cox proportional hazard regression analysis. Although lymph node status was statistically significant in progression-free survival analysis in univariate Cox proportional hazard regression analysis, lymph node status was not included in multivariate regression analysis due to unknown pathological status of lymph nodes in 9 patients. According to the clinical situation, pathologic grade and recurrent tumor that may affect the prognosis of bladder cancer were also included in the multivariate Cox proportional hazard regression analysis. Further multivariate Cox proportional hazard regression analysis was shown in Table 2. The results showed that TIPE2 was a protective factor for progression-free survival (*p* = 0.031), pT stage (*p* = 0.016) was a risk factor for progression-free survival, and age was a risk factor for overall survival (*p* = 0.020). Taken together, these data demonstrate that TIPE2 expression may serve as a biomarker for unfavorable outcome in patients with bladder cancer.

**TABLE 2:** Multivariate Cox proportional hazard regression analysis for predicting recurrence and survival of urothelial bladder carcinoma.

	PFS			OS		
	Z	HR (95%CI)	P	Z	HR (95%CI)	P
TIPE2 expression	−0.812	0.444 (0.212–0.928)	**0.031**	-0.763	0.466 (0.179–1.214)	0.118
Pathologic grade	−0.217	0.805 (0.240–2.702)	0.725	−0.191	0.826 (0.158–4.319)	0.821
pT stage	1.490	4.439 (1.323–14.894)	**0.016**	1.612	5.014 (0.976–25.755)	0.053
Age	0.029	1.029 (0.994–1.065)	0.101	0.055	1.057 (1.009–1.107)	**0.020**
Recurrent tumor	−0.166	0.847 (0.385–1.863)	0.680	−1.033	0.356 (0.099–1.276)	0.113

Z, regression coefficient; CI, confidence interval; HR, hazard ratio.

*p* values less than 0.05 are bolded.

## Discussion

Urothelial carcinoma is the most common histological type of bladder cancer. According to the depth of invasion, non-muscular invasive bladder cancer (NMIBC) and muscular invasive bladder cancer (MIBC) were classified. NMIBC patients are usually treated by transurethral resection of bladder tumor (TURBT), but about 24%–84% of NMIBC patients have recurrence within 5 years after surgery, and a considerable number of patients develop from NMIBC to MIBC. Radical cystectomy combined with pelvic lymph node dissection is used for the treatment of MIBC and high-risk NMIBC patients [18]. The 5-year survival rate of MIBC patients is around 50% [19], and nearly half of MIBC patients have micrometastasis at the time of diagnosis. The death of most cancer patients is related to tumor metastasis, among which lymphatic metastasis is the major route for cancer spreading of bladder cancer. The biological behavior of bladder cancer is complicated and volatile. At present, the diagnosis and postoperative surveillance mainly rely on invasive cystoscopy combined with imaging examination. Existing related biomarkers and prognostic models are inefficient in predicting the aggressiveness of the bladder cancer. Therefore, novel biomarkers are urgently needed to improve the accuracy of predicting tumor progression and patient prognosis.

Epithelial-mesenchymal transition (EMT) is considered as the initial step of tumor metastasis. EMT refers to the biological process by which epithelial cells are transformed into mesenchymal phenotypes by specific mechanisms. The process of EMT causes epithelial cells to lose apical and basal polarity and intercellular connectivity, leading to cytoskeletal rearrangement, mesenchymal morphology and increased mobility [20]. EMT has been demonstrated to be closely associated with the occurrence and development of bladder cancer by elevating the expression of EMT-related markers and activating EMT signaling pathway [21, 22].

TIPE2 has been primarily identified as an immunomodulator, but has also been shown to be expressed in a variety of non-immune tissues, such as glandular epithelium and squamous epithelium [23]. Several studies have shown that TIPE2 can participate in the EMT process of diseases through signaling pathways such as Wnt/β-catenin. For example, TIPE2 inhibits hypoxia-induced activation of the Wnt/β-catenin pathway in glioma [24]. TIPE2 overexpression inhibits gastric cancer metastasis by promoting β-catenin degradation and inhibiting β-catenin signaling pathway [25]. TIPE2 also could inhibit esophageal cancer by blocking the Wnt/β-catenin pathway [11]. In addition, studies have shown that TIPE2 expression is down-regulated in adenomyosis, suggesting that TIPE2 plays an important role in inhibiting the migration and invasion of endometrial cells. Further studies have found that TIPE2 could directly bind to β-catenin to reverse the occurrence of EMT and suppress metastasis of endometrial cells [26]. The tumor microenvironment of bladder cancer contains a large number of immune cells and is characterized by inflammation and immune suppression [27]. The EMT process also plays a significant role in the regulation of immune cells in the tumor microenvironment, and more and more evidence indicates that this regulation may be a key mechanism to promote the immune escape of cancer [28]. As a negative regulator of inflammation and immune function, TIPE2 is involved in tumorigenesis and development through a variety of signaling pathways, and its detailed function in cancers remains largely unknown. Therefore, we hypothesized that TIPE2 may play an important role in the development and progression of bladder cancer. The present study analyzed the expression and significance of TIPE2 in bladder cancer tissues by retrospective data and IHC staining. The results clearly showed that TIPE2 was expressed in urothelial carcinoma of bladder (105 of 110 cases were positive). And we found that TIPE2 protein was expressed in both the nucleus and cytoplasm of tumor cells. This different expression pattern of TIPE2 in the tumor cells may be related to different clinical features, which need to be further validated. Moreover, our analysis results indicated that TIPE2 could differentiate tumor metastasis and prognosis. These findings suggest that TIPE2 may be a potential biomarker in bladder cancer, and TIPE2 staining can be used to assess the risk and prognosis of tumor metastasis.

Finally, we acknowledge the limitations in our study. First, this study is a single-center retrospective study, so it may have selection bias. Second, due to the limitations of IHC method, tumor sampling bias may have partially influenced the results, although slides were analyzed separately by different pathologists and double-blind scoring was used to reduce this problem. Third, the follow-up time of our study is relatively short. Therefore, the relationship between the expression of TIPE2 and PFS or OS needs to be verified by a large cohort of patients. Fourth, TIPE2 expression in tumor cells or tumor microenvironment may have different effects on the prognostic outcome of patients. Finally, we need to assess the prognostic value of cytoplasmic versus nuclear TIPE2 expression in bladder cancer.

## Conclusion

This study systematically analyzed the expression characteristics, clinicopathological features and prognosis of TIPE2 in bladder urothelial carcinoma. Taken together, the results indicate that TIPE2 can be used as a biomarker for the disease progression and prognosis of patients with bladder urothelial cell carcinoma.

## Data Availability

The original contributions presented in the study are included in the article/Supplementary Material, further inquiries can be directed to the corresponding author.

## Supplementary Material

Supplementary Table S1 Univariate Cox proportional hazard regression analysis for predicting recurrence and survival of urothelial bladder carcinoma.
