# Supplementary material for: A Novel Prognostic Factor TIPE2 in Bladder Cancer
Source: Pathol Oncol Res. 2022 Mar 21;28:1610282. doi: 10.3389/pore.2022.1610282 (PMC8978781; doi:10.3389/pore.2022.1610282)
Supplement: Supplementary file 1 [file Table1.doc]

**Supplementary Table 1: Univariate Cox proportional hazard regression analysis for predicting recurrence and survival of urothelial bladder carcinoma**

|  | **PFS** | | | **OS** | | |
| --- | --- | --- | --- | --- | --- | --- |
|  | **Z** | **HR(95%CI)** | **P** | **Z** | **HR(95%CI)** | **P** |
| **TIPE2 expression** | -0.778 | 0.459(0.225-0.940) | **0.033** | -0.546 | 0.579(0.233-1.440) | 0.240 |
| **Pathologic grade** | 0.921 | 2.511(0.880-7.167) | 0.085 | 1.057 | 2.877(0.664-12.467) | 0.158 |
| **pT stage** | 1.526 | 4.601(1.613-13.124) | **0.004** | 1.670 | 5.313(1.227-23.000) | **0.025** |
| **Age** | 0.040 | 1.041(1.005-1.077) | **0.023** | 0.067 | 1.070(1.020-1.122) | **0.006** |
| **Recurrent tumor** | -0.034 | 0.966(0.447-2.089) | 0.931 | -0.878 | 0.416(0.121-1.427) | 0.163 |
| **Lymph node status** | 0.749 | 2.114(1.061-4.211) | **0.033** | 0.139 | 1.150(0.482-2.740) | 0.753 |
| **Gender** | 0.209 | 1.232(0.507-2.995) | 0.645 | -0.028 | 0.972(0.283-3.337) | 0.964 |
| **Vascular invasion** | 0.257 | 1.293(0.775-2.157) | 0.325 | 0.168 | 1.183(0.610-2.294) | 0.619 |
| **Nerve invasion** | 0.017 | 1.017(0.640-1.615) | 0.942 | 0.299 | 1.348(0.702-2.587) | 0.369 |

Z=regression coefficient; CI = confidence interval; HR = hazard ratio.
